# Prescriptions and Dispensing

**Published:** 1908-08-22

**Authors:** 


### Therapeutics.

### PRESCRIPTIONS AND DISPENSING.

### POWDERS (*continued*).

Rare though reaction between dry powders may be, certain solid substances, when mixed even in the solid state, come slowly into reaction or combination, and sometimes a liquid is the result. The commonest example is, perhaps, that of chloral and camphor. There are several substances among the higher alcohols and phenols, such as menthol, thymol, and so forth, which give similar results. Probably, however, the only substances which show this behaviour that are likely to be ordered together as powders are antipyrine and a salicylate. Antipyrine and sodium salicylate, for example, may inadvertently be prescribed together as a powder; when mixed, they form a liquid which contains salicylate of antipyrine. When one is familiar with this fact, one will probably prescribe antipyrine salicylate in the form of a liquid mixture; but if it is for some reason or other important to have the medicine in powder form, the only plan is to dispense the incompatibles separately, giving the patient instructions to mix together one of each at the time of taking.

The same procedure is necessary in the case of Seidlitz powders, since, although the sodium bicarbonate and the tartaric acid of the latter would not react so long as the powders were kept absolutely dry, the formation of sodium tartrate and the evolution of $CO_2$ gas would begin in the presence of the least dampness.

Many powders have a tendency to absorb moisture and become damp; some are absolutely deliquescent —calcium chloride, for example. If it is desired to give these in the form of powder rather than as a mixture, they must be protected from the air. They may be sealed in cachets or capsules, for example; or the ingredients may be weighed out in an absolutely dry state, and each powder may then be wrapped in a waxed paper which is sealed up.

When it is desired to give a mixture which shall be in a state of effervescence when taken by the patient, it is usual for one of the ingredients to be a bicarbonate, tartaric or citric acid being ordered in powders, one of which is to be mixed with each dose; or the mixture may contain the acid, and the bicarbonate may be prescribed as the powder that is to be mixed in and stirred at the time of taking. In other cases two mixtures are ordered, one acid and the other containing the bicarbonate, a certain portion of each to be mixed and stirred before taking. The following are two examples of prescriptions in which a powder is added to the mixture to make an effervescing dose:—

| | | |
|---|---|---|
| ℞ Tincturæ Asafœtidæ | ... ... ... | ʒss. |
| Mucilaginis Acaciæ | ... ... ... | ʒss. |
| Tincturæ Valerianæ Ammoniatæ | ... | ʒss. |
| Ammonii Carbonatis | ... ... ... | gr. xx. |
| Aquam | ... ... ... ... ad | ℥j. |

Misce. Mitte doses viij.

Acidi Citrici ... ... ... ... gr. xv.

Mitte pulveres viij.

Sig.: One of the powders to be mixed with two tablespoonfuls of the mixture, stirred, and the medicine to be taken in an effervescing state.

| | | |
|---|---|---|
| ℞ Liquoris Strychninæ | ... ... ... | ♏xl. |
| Syrupi Limonis | ... ... ... ... | ʒiij. |
| Acidi Citrici | ... ... ... ... | ʒij. |
| Aquam | ... ... ... ... ad | ℥vj. |

Sig.: $\frac{1}{6}$ pro dose, cum pulvere uno, in statu effervescente sumendo.

℞ Sodii Bicarbonatis ... ... ... gr. xxiv.
Fiat pulvis j. Mitte vj.

The sodium bicarbonate is given as the separate powder, instead of the citric acid, in the latter prescription, because if the soda were in the mixture it would react with the syrup of lemon, and would also be liable to precipitate the strychnine with consequences that might be dangerous to the patient.

Dusting powders are, of course, not subdivided, but dispensed in bulk. Essential oils may be ordered in them in small quantities. These should be added to the most absorbent ingredient, or to a small portion of it, and then further quantities of the powder may be gradually mixed in until there is no longer any appearance of dampness. Liquid of any sort should never be added to the whole bulk of mixed powder.

Dusting powders should be subjected to a final sifting, either through a fine-meshed metal sieve, or through very fine muslin. A piece of muslin stretched across a chip-box, from which the bottom has been removed, makes a convenient sieve for small quantities of powder. It is a good plan to sift any powder in which the nature of the ingredients makes extra mixing desirable.

Insufflations and Snuffs are for the most part prepared in the same way as other powders are. Sometimes special methods may be necessary, as in the following prescription:—

| | | |
|---|---|---|
| ℞ Tincturæ Benzoini Simplicis | ... ... | ʒj. |
| Acidi Borici ... | ... ... ... ... ... | ʒj. |
| Pulveris Amyli | ... ... ... ... | ʒj. |

Fiat pulvis pro insufflatione ut dictum.

The two powders should be well mixed, the tincture then added, and stirred in a small quantity at a time, and the whole exposed in a flat dish in a moderately warm place until the alcohol has evaporated. When dry, the mixing may be made more thorough still by further trituration in the mortar, together with occasional mixing by means of a spatula on paper.

Powders of this kind are usually sent out in bulk, but it would be quite easy, of course, to sub-divide into doses one powder at a time, to be used in the insufflator or as snuff as directed.

Powders are often put into cachets to facilitate their being swallowed by the patient. A cachet is a hollow receptacle, consisting chiefly of rice flour; when dry it is stiff and brittle, but when just dipped into water it becomes very soft without bursting, so that it is easily swallowed without the contents coming into contact with the tongue. Each cachet is made in two halves, so that filling is easy. One half is laid upon the table, the dose of powder is poured into it through a small funnel; the rim of the other half is just moistened with water, applied to the half that has been filled, and gently pressed down. The two halves adhere, and the moistened rims are soon dry. Cachets are made of various sizes. The smallest size compatible with holding the powder required should always be employed.

(*To be continued.*)

